# Isolated ventricular noncompaction: a case report

**DOI:** 10.1186/1757-1626-2-9312

**Published:** 2009-12-11

**Authors:** João Bento, Filipe Monteiro, Luis Sargento, Joaquin Vizcaino, Jorge Monteiro, Pilar Azevedo, Gabriela Brum

**Affiliations:** 1 Pulmonology Department, Hospital S João, Alameda Professor Hernâni Monteiro 4300-319, Porto, Portugal; 2 Respiratory Intensive Care Unit, Pulmonology Department, Hospital Santa Maria, Avenida Professor Egas Moniz, Cidade Universitária, 1649-028 Lisboa, Portugal; 3 Lisbon Medical Faculty, Avenida Professor Egas Moniz (Hospital Santa Maria), Cidade Universitária, 1649-028, Lisboa, Portugal; 4 Cardiology Department, Hospital Santa Maria, Avenida Professor Egas Moniz, Cidade Universitária, 1649-028 Lisboa, Portugal

## Abstract

Isolated ventricular noncompaction is an extremely rare cardiomyopathy, not fully clarified.

It is characterized by persistent embryonic myocardium morphology without associated cardiac abnormalities.

Since first description in 1984, few clinical studies were done. Data in the literature are lacking and most reports consist on a few case studies.

Doppler ecocardiogram is considered the reference method for diagnosis.

Diagnosis remains difficult since there are similarities with other cardiac defects, clinical manifestations are non-specific and echocardiographic criteria are not universally accepted.

As a consequence diagnosis may be easily missed.

Moreover, clinical and echocardiographic features were just recently clarified.

Treatment is directed towards important clinical manifestations (heart failure, arrhythmias and embolic events).

We present a clinical case of severe cardio-respiratory failure in previously healthy and asymptomatic young male, which was the initial presentation of an isolated ventricular noncompaction.

A brief review of available literature is done concerning to this case study.

## Background

Isolated ventricular noncompaction (IVNC) is a rare cardiomyopathy, until now, not fully clarified. It is thought to result from an arrest of the compaction of loose myocardial meshwork during foetal development. It is generally associated with other congenital abnormalities such as obstruction of ventricular outflow tracts.

IVNC is characterized by persistent embryonic myocardium morphology in the absence of other cardiac abnormalities.

Engberding first described it in 1984 referring to a 33-year old woman with persistent "sinusoids" in the left ventricle as an isolated abnormality [1]. Since then few clinical studies have been done on relatively small number of patient's cohorts. Most of the literature is based on a few case reports. Due to lack of pathophysiological characterisation, IVNC has been unspecifically assigned to a heterogeneous group of "unclassified cardiomyopathies". As a consequence, diagnosis is frequently missed, with important negative prognostic implications for these patients.

## Case presentation

Thirty-two years old, black male, born in Angola, mason and smoker. He was previously healthy, referring no prior cardiac or pulmonary complaints. He had a two-month history of asthenia, anorexia and mild to moderate progressive dyspnoea with a decreased exercise tolerance. About 72 hours before his admission to the hospital, he started with fever, cough, mucopurulent sputum, severe shortness of breath and total exercise intolerance.

**Figure 1:**
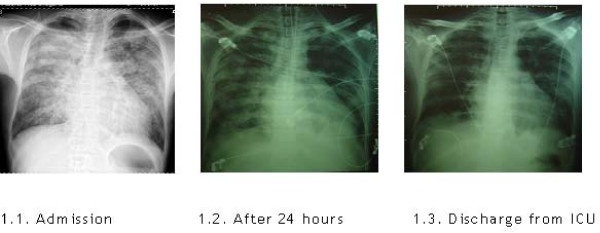
**Chest X-ray evolution (admission to discharge from ICU)**. Increased heart-thorax index and heterogeneous diffuse infiltrate sparing upper lobes and bases. Favorable radiological evolution.

On admission to the Emergency Ward he was on acute pulmonary oedema with respiratory failure and acidosis. Chest x-ray demonstrated increased heart-thorax index and heterogeneous diffuse infiltrate sparing the upper lobes and bases. (Figure 1)

He was referred to Intensive Care Unit (ICU) where he was intubated and submitted to mechanical ventilation. He was prescribed with antibiotics and diuretics.

A former bedside echocardiogram was performed, six hours after admission, with the patient under ventilator support, showing mild mitral valve regurgitation. No additional abnormalities were found and left ventricle function was normal.

The B-type natriuretic peptide (BNP) levels were 2152 pg/mL (normal value ≤ 88 pg/mL).

Microbiological and immunological studies were negative.

Patient presented an adequate clinical response and on 5^th ^day of mechanical ventilation he was extubated.

Two more episodes of acute pulmonary oedema occurred which were reverted by medical therapy and non-invasive ventilation.

He started on angiotensin-converting enzyme (ACE) inhibitors therapy.

**Figure 2:**
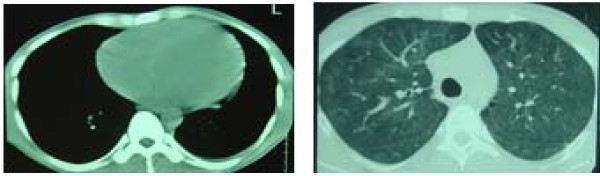
**Chest axial tomography**. Cardiomegaly, bilateral basal patchy ground glass opacities in resolution process.

Chest axial tomography (Figure 2) showed cardiomegaly and bilateral basal patchy ground glass opacities in resolution process.

**Figure 3:**
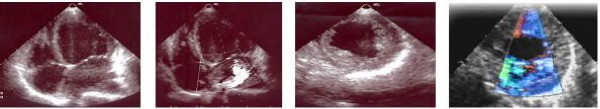
**Echocardiogram suggesting IVNC**. Exuberant thickening and trabeculation of left ventricle(LV) apical wall. Ratio between non-compacted endocardium and compacted epicardium = 13/6 (measured at end systole in parasternal short axis view). Dilated LV, diffuse hypokinesis, ejection fraction = 38%. Colour Doppler image showing recesses supplied by intraventricular blood.

A new echocardiogram (Figure 3) demonstrated exuberant thickening and trabeculation of the lateral and posterior walls of the apical half of the left ventricle with two distinct myocardial layers: a normal compact (C) epicardium and a thickened non-compact (NC) endocardium. The ratio between NC endocardium and C epicardium = 2,2 (measured at end systole in parasternal short axis view). Left ventricle cavity was dilated and presented diffuse hypokinesis and an ejection fraction of 38%. No additional abnormalities were found. These findings were consistent with the diagnosis of IVNC.

After nine days on the ICU, patient was clinically stabilized and was referred to a Cardiology ward. BNP although far beyond normal levels, had decreased to 1244 pg/ml.

**Figure 4:**
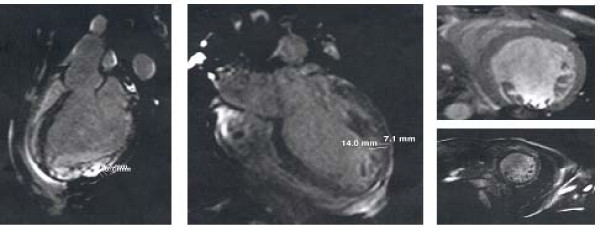
**Cardiac Magnetic Resonance Imaging confirming IVNC**. Numerous proeminent trabeculae and deep intertrabecular recesses. Ratio between distance from the epicardial surface to the trough of the trabecular recess/distance from the epicardial surface to peak of trabeculation = 6,7/19,2 (left image). Two-layered myocardium, ratio non-compacted endocardium to compacted epicardium = 2 (middle image).

A cardiac Magnetic Resonance Imaging (MRI) (Figure 4) was performed which confirmed the diagnosis, revealing numerous proeminent trabeculae and deep intertrabecular recesses and a ratio between the distance from the epicardial surface to the trough of the trabecular recess/distance from the epicardial surface to peak of trabeculation = 0,35.

A β-blocker was added to the treatment already prescribed and patient was discharged on the 15^th ^day after admission and referred to a Cardiology clinic.

## Discussion and bibliographic review

IVNC is considered a rare disease, although its exact prevalence of is unknown. According to one of the largest series published, its prevalence was 0,014% in a group of patients referred to an echocardiography laboratory for abnormal findings or congestive heart failure [2]. Men appear to be more frequently affected, accounting for 56 to 82% of reported cases [2,3].

The initial series published were related to paediatric population, but more recently there have been some cases described in adults [1-4].

IVNC is considered a congenital cardiomyopathy caused by intrauterine arrest of myocardial development. During early embryonic development, in the first months of intrauterine life the myocardium consists of a meshwork of loosely interwoven muscle fibres. In this spongy conformation, trabeculae alternate with recesses that communicate with the ventricular cavity to provide blood supply to the myocardium. Between the 5^th ^and the 8^th ^week of intrauterine development, when coronary circulation develops, ventricular myocardium becomes gradually compacted and those recesses turn into capillaries [3,5].

Left ventricle is uniformly affected, while right ventricle involvement is referred in less than one-half of the patients [3].

Familial recurrence has been associated to genetic linkage, related to a mutation in G 4.5 gene of Xq28 chromosome region and to a cardiac specific CSX gene [2].

Clinical manifestations of IVNC are non-specific, heterogeneous and patients may be either asymptomatic or suffer from severe cardiac dysfunction. Although it is a congenital disease, the onset of symptoms is quite variable and may even occur at an advanced age. One of the most common manifestations of IVNC is a progressive left ventricular dysfunction, which may cause heart failure.

Structural myocardial changes, consequent microvascular dysfunction and subendocardial perfusion defects, even in the absence of coronary disease, may contribute either to ventricular dysfunction or arrhythmias [2,3,5,6]. Atrial fibrillation and ventricular tachyarrhythmias are the most frequent arrhythmias described. Sudden cardiac arrest occurred in about 47% of patients with IVNC [2-4]. Other electrocardiographic abnormalities found in most patients include left ventricular hypertrophy, ST segment changes, inverted T wave, intraventricular conduction defect and AV block [2,3,5,6].

Embolic events resulting either from atrial fibrillation or from formation of thrombi in the ventricular trabeculae may occur quite frequently. Cerebrovascular accidents, transient ischemic attacks and mesenteric infarction related to IVNC have been described in the literature [2,3,5].

IVNC is generally detected by means of bidimensional echocardiography with Doppler, which is considered the reference method for the diagnosis [5,7,8]. Echocardiographic findings consist on a two-layered myocardium with a thin (compacted) epicardium, a thick (non-compacted) endocardium composed of a trabecular meshwork, numerous proeminent trabeculae and deep intertrabecular recesses in continuity with ventricular cavity. Predominant segmental locations occur in the mid lateral, apical and inferior walls. Hypokinetic movements occur either in the affected areas or in the surrounding normal segments [7]. No other abnormalities should be found [2,7-9].

Cardiac MRI has good correlation with echocardiogram, being useful in patients with bad echocardiographic window and to clarify doubtful echocardiographic abnormalities [5,8].

Diagnosis of IVNC remains sometimes difficult and even controversial. IVNC often presents overlapping findings to other heart muscle diseases like dilated cardiomyopathy (DCM) and hipertrophic cardiomyopathy (HCM) [7]. It's not recognized as an independent and well defined entity and many authors even prefer the term hypertrabeculation instead of IVNC. So, IVNC is a primary genetic cardiomyopathy, which diagnostic criteria are not universally accepted and is still considered in the unclassified group of cardiomyopathies [10,11]. As a consequence, recognition and diagnosis may be delayed which compromises its correct management.

This clinical case refers to a previously healthy black male presenting an acute severe cardio-respiratory failure requiring ICU admition. Both echocardiogram and cardiac RMI were suggestive of IVNC. No other structural abnormalities or cause for acute heart failure were detected.

Several diagnostic criteria have been proposed in the literature [2,7-9,12,13]. In this clinical case diagnosis was primarly established by echocardiogram according to the criteria defined by *Jenni and colleagues *[7]. According to these authors diagnosis is based on the detection of 2 myocardial layers, with a normal compact (C) epicardium and a thickened non-compact (NC) endocardium. They propose a quantitative evaluation by determining the ratio between the 2 layers NC/C ≥ 2, measured at end systole in parasternal short axis view, to establishe diagnosis [7]. This allows differentiation of the trabeculations of IVNC from that observed with DCM and HCM [7].

This clinical case also meets the Chin diagnostic criteria which are based in the ratio between distance from the epicardial surface to the trough of the trabecular recess/distance from the epicardial surface to peak of trabeculation ≤ 0,5 [13].

Both images obtained by echocardiogram and cardiac RMI were suggestive of IVNC.

Meanwhile recently *Kohli and colleagues *have proposed that diagnostic criteria of IVNC may be too sensitive for black individuals [12]. They refer that ecocardiographic findings suggestive of IVNC may represent either a genuine congenital abnormality or an exaggeration of normal trabeculation pattern [12]. In fact, their series makes reference to normal black individuals fulfilling criteria of IVNC that present normal or subtle clinical phenotype, which is not the case of this clinical report [12].

Extensive non compacted myocardial layer can be just a variant of normal maturation of the ventricular wall. The most important is to determine when its presence becomes clinically relevant rather than an incidental finding. In fact to decide the clinical significance of these findings in an asymptomatic individual with no additional findings may be a huge dilemma.

Treatment of IVNC is not specific and is directed toward its three most important clinical implications: heart failure, cardiac arrhythmias and embolic events.

Appropriate treatment for heart failure is essential and is similar to that of other causes which include diuretics, β-blockers and ACE inhibitors [3,5,8]. If standard treatment for heart failure is unsuccessful, heart transplantation may be considered as the only therapeutic possibility [3,5,8]. Some authors suggest that assessment of arrhythmias by electrophysiologic study and 24-hour Holter ECG monitoring should be performed at the initial assessment and then annually [5]. For patients with arrhythmia, either symptomatic or not, anti-arrhythmic drugs are recommended. Some authors have used a biventricular pacemaker [5,9]. Attending to the risk of cardiac sudden death, implantable cardioverter-defibrillator (ICD) may have a role in these patients [5,9]. Long-term prophylactic anticoagulation is recommended for all patients with IVNC regardless the identification of intracardiac thrombus [2,3,5,8].

Sports competition and exhausting activities are forbidden and pregnancy should be avoided [5]. Due to familial association of IVNC, first-degree relatives of patients should undergo echocardiography as a screening test [5].

Prognosis is widely variable. Some patients remain asymptomatic throughout their lives, while others show a rapid deterioration of the cardiac function, causing early death [5,7]. In fact, about 47-60% of symptomatic patients require heart transplantation in the first 6 years after diagnosis [2,3,5,8]. Asymptomatic patients, identified through routine or screening echocardiographic evaluation, have a clearly better prognosis [3]. Enlarged left atrium, left ventricular cavity dilatation, symptomatic heart failure class III-IV *New York Heart Association (NYHA)*, persistent ventricular tachycardia, atrial fibrillation and branch block are bad prognosis criteria [2,5,9]. These high-risk patients have unstable clinical course and should be treated aggressively including early consideration of implantable cardioverter-defibrillator (ICD) or heart transplant.

As previously referred diagnosis and orientation of IVNC patients with relevant clinical presentation is usually difficult and complex.

However not easier is the approach of asymptomatic individuals in which a routine echocardiogram reveals extensive hypertrabeculation or even suggests IVNC. These problems may surely increase as cardiac image techniques will continue to improve and until more accurate criteria become available.

There is much work to be done in order to clarify this entity with such a heterogeneous spectrum. Additional studies are needed to determine the frontier between upper limits of normal trabecular patterns that may occur in asymptomatic individuals and clinically relevant IVNC.

## Conclusion

Isolated ventricular noncompaction is a rare and recently described congenital cardiomyopathy. Its impact on morbidity and mortality is gaining importance as it is recognized with increasing frequency. An early, reliable diagnosis is crucial and echocardiography represents a prominent role.

Treatment is directed towards its most important clinical implications, which include heart failure, arrhythmias and systemic embolic events. Heart transplantation is the only definitive treatment.

Our purpose with this case report was to point out attention to this entity as an important and often misdiagnosed cause of cardio-respiratory failure.

## Abbreviations

IVNC: Isolated ventricular noncompaction; ICU: Intensive Care Unit; BNP: B-type natriuretic peptide; DCM: Dilated cardiomyopathy; HCM: Hipertrophic cardiomyopathy

## Consent

Written informed consent was obtained from the patient for publication of this case report and accompanying images.

A copy of the written consent is available for review by the Editor-in-Chief of this journal.

## Competing interests

The authors declare that they have no competing interests.

## Authors' contributions

JB, FM, JV, JM, PA, GB were staff from the ICU. All of them analyzed and interpreted the patient data regarding to the clinical presentation, complementary exams. They were responsible for the orientation of this clinical case in the ICU. They were major contributors in writing and reviewing the manuscript. LS performed the cardiological examination. He was responsible for cardiological orientation after discharge from ICU. All authors read and approved the final manuscript.
